# Gamma-linolenic acid ameliorated glycation-induced memory impairment in rats

**DOI:** 10.1080/13880209.2017.1331363

**Published:** 2017-05-26

**Authors:** Shahab Ali Khan, Ali Haider, Wajahat Mahmood, Talat Roome, Ghulam Abbas

**Affiliations:** a Department of Pharmacy, COMSATS Institute of Information Technology, Abbottabad, K.P.K., Pakistan;; b Department of Pathology, Dow International Medical College, Dow University of Health Sciences, Karachi, Pakistan;; c Pharmacology Section, H.E.J. Research Institute of Chemistry, International Center for Chemical & Biological Sciences, University of Karachi, Karachi, Pakistan

**Keywords:** Advanced glycation end products, ageing, learning and memory

## Abstract

**Context:** γ-Linolenic acid (GLA) is an important constituent of anti-ageing supplements.

**Objective:** The current study investigates the anti-ageing effect of GLA in Sprague-Dawley rats.

**Materials and methods:** GLA (0.1, 0.2, 0.4, 2, 10, 20 and 24 μM) was initially evaluated for its effect on the formation of advanced glycation end products (AGEs) *in vitro*. For *in vivo* assessment (1, 5 or 15 mg/kg), the rat model of accelerated ageing was developed using d-fructose (1000 mg/kg (i.p.) plus 10% in drinking water for 40 days). Morris water maze was used to evaluate impairment in learning and memory. The blood of treated animals was used to measure glycosylated haemoglobin (HbA1c) levels. The interaction of GLA with active residues of receptor of AGE (RAGE) was analyzed using AutoDock Vina.

**Results:** Our data showed that GLA inhibited the production of AGEs (IC_50_ = 1.12 ± 0.05 μM). However, this effect was more significant at lower tested doses. A similar pattern was also observed in *in vivo* experiments, where the effect of fructose was reversed by GLA only at lowest tested dose of 1 mg/kg. The HbA1c levels also revealed significant reduction at lower doses (1 and 5 mg/kg). The *in silico* data exhibited promising interaction of GLA with active residues (Try72, Arg77 and Gln67) of RAGE.

**Conclusion:** The GLA, at lower doses, possesses therapeutic potential against glycation-induced memory decline.

## Introduction

Ageing leads to progressive impairment in cognition, particularly learning and memory (Evans et al. 1984; Nagy & Aubert 2015). The non-enzymatic glycation theory states that the levels of advanced glycation end products (AGEs) increase with age, which leads to decline in cellular functions (Monnier 1988; Brownlee 1995; Jono et al. 2002; Yaffe et al. 2011). AGEs are the structurally modified proteins or lipids produced by non-enzymatic process termed as glycation. The critical factors important for the AGE formation are the turnover rate of proteins for glycoxidation, blood glucose levels (hyperglycaemia) and the environmental stress (Goldin et al. 2006). The deleterious effects of AGEs are partly mediated *via* receptor for advanced glycation end products (RAGEs) (Sheetz & King 2002; Ahmed 2005). Briefly, the interaction of AGEs with RAGE triggers the activation of the nuclear factor kappa B (NF-κB) followed by the activation of several pro-inflammatory cytokines like tumour necrosis factor-α (Kalousova et al. 2002; Sheetz & King 2002; Basta et al. 2004; Bierhaus et al. 2005; Lukic et al. 2008). This in turn causes oxidative stress and leads to decline in bodily faculties.

A review of the literature revealed that high carbohydrate intake leads to increase in the formation of AGEs because more substrate is available for glycation. In this regard, fructose is reported to enhance the production of AGEs (Farooqui 2013) and presumably memory impairment in humans (Yaffe et al. 2011; West et al. 2014). In animals, the large doses of fructose have also been reported to escalate the glycation, thereby leading to the development of associated anomalies (Rodriguez et al. 1994).

GLA is a polyunsaturated fatty acid having 18-carbon chain with three double bonds at 6th, 9th and 12th position (Huang & Ziboh 2001). It is present in various vegetable oils including hemp oil and borage oil. In mammals, it is synthesized from linoleic acid (dietary) with the help of δ-6-desaturase enzyme. After formation, GLA is elongated to dihomo-γ-linolenic acid (DGLA) by an elongase enzyme. δ-Desaturase enzyme acts on DGLA and converts it to arachidonic acid (AA). Both DGLA and arachidonic acid are metabolized producing eicosanoids (Huang & Ziboh 2001). Epidemiological data revealed that the consumption of polyunsaturated fatty acids is beneficial for memory and cognition (Kalmijn et al. 1997, 2004). GLA is an important constituent of anti-ageing supplements. It has been reported to possess various biological effects including improvement of age-linked anomalies (Knauf et al. 2011). Keeping this in mind, the present study was aimed at investigating the effect of GLA on AGE modulation and associated memory impairment.

## Materials and methods

### Animals

Sprague-Dawley rats (150–200 g) were obtained from the Animal Care Facility of the COMSATS Institute of Information and Technology, Abbottabad. The animals were kept under standard condition of temperature (25 ± 1 °C) and 12-h light/dark cycle with free access to food and water. All experiments were performed according to the guidelines of the ethical committee of CIIT, conforming to the guidelines of Animal Scientific Procedure Act 1986 (UK).

### Chemicals

The chemicals used in the study were as follows: aminoguanidine HCl and GLA (Santa Cruz Biotechnology, Dallas, TX), d-fructose (BDH Laboratory Supplies, Poole, England), bovine serum albumin (BSA), sodium dihydrogen phosphate and sodium hydrogen phosphate (Sigma Aldrich, St. Louis, MO).

### *In vitro* study

The effect of GLA on AGE formation was investigated (*in vitro*) as described previously (Vinson & Howard 1996). To make the AGE reaction mixture, BSA solution (10 mg/mL) was prepared in 0.2 M sodium phosphate buffer and added to fructose (100 mM). Various dilutions (0.5, 1, 2, 10, 50, 100 and 120 μM) of GLA were prepared and mixed with the BSA–fructose mixture. The reaction mixtures were incubated for 7 days. BSA solution was used as blank while aminoguanidine (2 μM) served as positive control. After incubation, 40 μL from each preparation was diluted (5×) with 0.2 M sodium phosphate buffer to get the final volume of 200 μL and was transferred to the black 96-well plate. Florescence intensity was measured at excitation and emission wavelength of 340 and 435 nm using spectrofluorometer FLUOstar Omega (BMG Labtech, Offenburg, Germany). Percent inhibition of AGE formation was calculated as follows:

%Inhibition=[1−(Fluorescence intensity of mixture with inhibitor)/(Fluorescence intensity of negative control mixture)]×100

### Treatment

Animals were divided into five groups of six animals each. Group 1 served as vehicle control and received 0.9% normal saline for 40 days. Group 2 animals (fructose group) were administered with d-fructose (1000 mg/kg (i.p.) plus 10% in drinking water for 40 days) (Jalal & Moghimi 2011). Groups 3, 4 and 5 along with fructose also received GLA (i.p.) at a dose of 1, 5 or 15 mg/kg body weight, respectively, for 40 days.

### Morris water maze

The Morris water maze (MWM) test was performed to assess the spatial navigation ability as described previously (Morris 1984). The MWM pool (black colour, 180 cm in diameter and 50 cm in height) was divided into four equal quadrants. A black colour circular platform, having a diameter of 10 cm and height of 20 cm, was kept in the centre of one of the quadrant for escape. Its position remained constant throughout the experiment. The temperature of water in the pool was maintained at 25 ± 2 °C throughout the experiment. Special distal cues were fixed around the wall of the pool to assist the animals in finding the escape platform. At 1st day (familiarization session), the platform was kept 1 cm above the water surface. In this session, five trials were performed to train the animals. The length of each trial was of 120 s. Rats were kept in one of the quadrant of the pool keeping its face towards the wall of the pool. If the animal found the platform within 120 s, it was allowed to stay on the platform for 5 s. In case animal failed to find the platform within 120 s, it was guided to the platform gently and was allowed to stay there for 30 s. On the 2nd, 3rd, 4th and 5th day (acquisition sessions), the platform was kept 1 cm below the water surface and five trials were conducted for each animal following the same procedure as for 1st day. After 24 h of the last acquisition phase, a probe trial was conducted to assess the retention of spatial memory by removing the hidden platform from the water pool. During probe trial, the rat was allowed to search the platform for 120 s. To eliminate the possible interrupting variables (like faecal matter, urine and stress odours), water pool was drained and refilled with fresh water on each day prior to the start of the experiment. A video camera was fixed to record the experiment. The videos were used to measure the escape latency, time taken to reach the target quadrant (the quadrant having the platform), time spent in the target quadrant and number of crossing through platform position in order to assess the spatial memory.

### Locomotor activity test

The locomotor performance of the rats was evaluated immediately following the probe trial of the MWM. The animals were kept in the 46 × 46 cm locomotor boxes divided into four equal quadrants. The animals were kept in the boxes for 6 min allowing the 1st min for habituation. The number of boxes crossed by the rats was counted and compared with that of the control.

### Estimation of HbA1c

Rats were sacrificed and blood was collected by cardiac puncture in blood collection tubes containing EDTA. Haemolyzing agent (1 mL, Lyser cell™ WDF, Sysmex America Sysmex America, Mundelein, IL) was mixed with blood (10 μL) for 5 min in reaction cuvettes followed by subjecting to Cobas Chemistry Autoanalyzer (Roche Diagnostics, Indianapolis, IN) for determination of HbA_1C_.

### *In silico* study

The structure of the ligand-binding domain of the receptor for AGEs (3CJJ) was downloaded from the Protein Data Bank (PDB) website (www.rcsb.org/pdb). The binding site residues of the receptors were identified by first docking N6-carboxymethyllysine (CML), an AGE, on to the structure of 3CJJ. The CML molecule was then removed from the receptor binding site using Discovery Studio 4.0. The receptor and ligand structures were then prepared for docking using AutoDock tools (Morris et al. 2009). GLA was then docked into the binding domains of AGE receptor using AutoDock Vina (Oleg & Arthur 2009). Nine binding modes were proposed by the program for each of the domains and the binding mode with lowest binding energy was selected and studied for interactions with the receptor molecule using Pymol (The PyMOL Molecular Graphics System, version 1.5.0.4, Schrödinger, LLC, New York, NY).

### Data analysis

All results are expressed as mean ± standard error of the mean (S.E.M) of six animals per group. The differences between various means were computed using one-way ANOVA followed by Dunnett’s test using statistical package SPSS 19.0 software (Chicago, IL). Throughout statistical analysis, *p* < 0.05 was considered to be statistically significant.

## Results

### *In vitro* AGE formation assay

**Figure 1.**
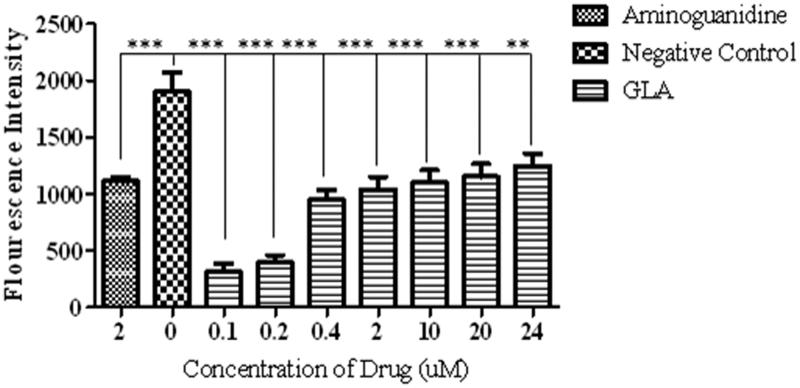
Effect of various concentrations of GLA on the formation of AGEs. The data are expressed as mean ± SEM (*n* = 6) of fluorescence intensity, which co-relates with the production of AGEs. The GLA (0.1, 0.2, 0.4, 2, 10, 20 and 24 μM) appeared to be more effective in reducing the AGE formation at lower doses. The positive control (aminoguanidine) also exhibited the decline in the production of AGEs.

In similarity with standard aminoguanidine, the GLA treatment significantly [*F*(8, 18) = 20.71, ***p < 0.001, **p < 0.01] reduced the formation of AGEs as reflected by decrease in fluorescence intensity as compared to negative control (Figure 1, IC_50_ = 1.12 ± 0.05 μM). However, the GLA appeared to be more effective in inhibiting AGE formation at low doses as shown by dose-dependent increase in fluorescent intensity.

### Morris water maze

#### Acquisition sessions

**Figure 2.**
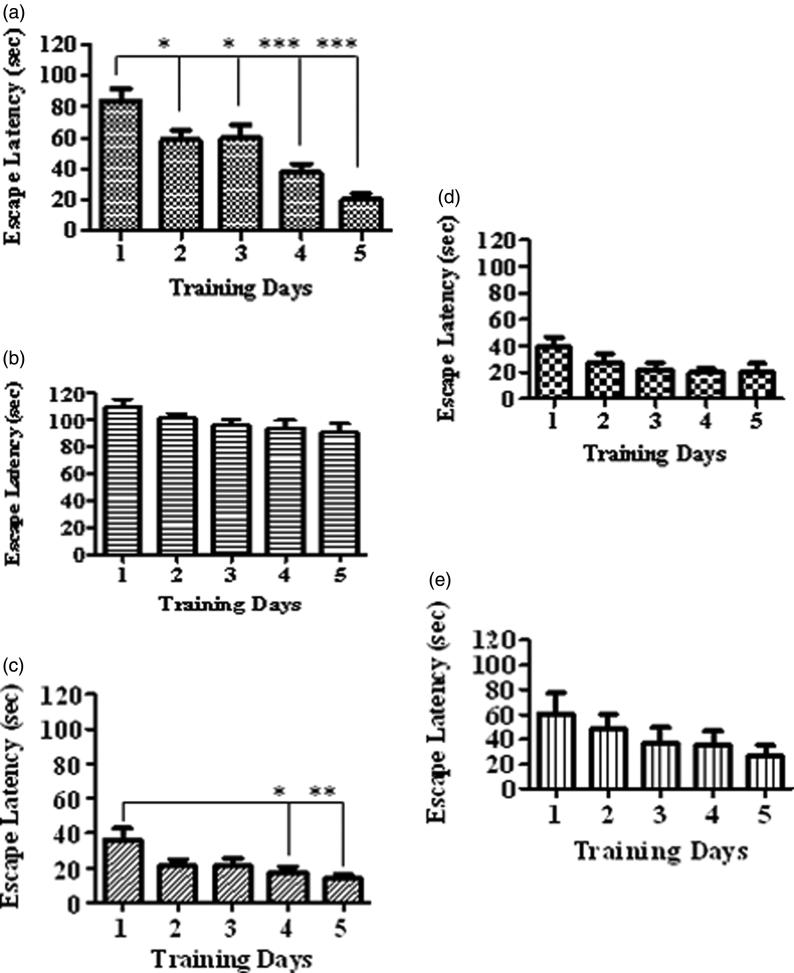
Comparison of escape latencies among various treatment groups during familiarization/acquisition trials in Morris water maze. The figure depicts the escape latency in a) vehicle control group, b) fructose group, c) GLA (1 mg/kg), d) GLA (5 mg/kg) and e) GLA (15 mg/kg). The vehicle group showed significant decline in the escape latency with the number of days, which is suggestive of the learning and memorization of spatial information. Among all groups, similar pattern was only observed in the GLA (1 mg/kg) group.

The control rats upon training showed significant decline in escape latency as compared to 1st day (Figure 2(a), [*F*(4, 20) = 14.47, *p < 0.05, ***p < 0.001]). However, the d-fructose treatment did not show significant learning during the progressive days (Figure 2(b)). The rats treated with GLA (1 mg/kg, Figure 2(c), [*F*(4, 25) = 4.03, *p < 0.05, **p < 0.01]) also displayed decrease in escape latency, while the effect was nonsignificant at doses of 5 and 15 mg/kg (Figure 2(d,e), respectively).

#### Probe trial

##### Time spent in the target quadrant

**Figure 3.**
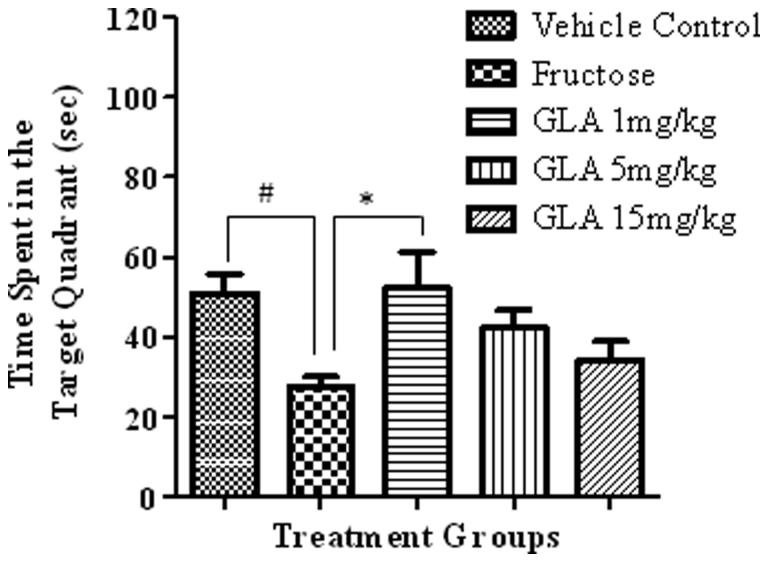
Effect of GLA on the time spent in the target quadrant during probe trial. The data are expressed as mean ± SEM (*n* = 6). The fructose treatment has significantly decreased the time spent in the target quadrant, which was significantly reversed by GLA at 1 mg/kg.

**Figure 4.**
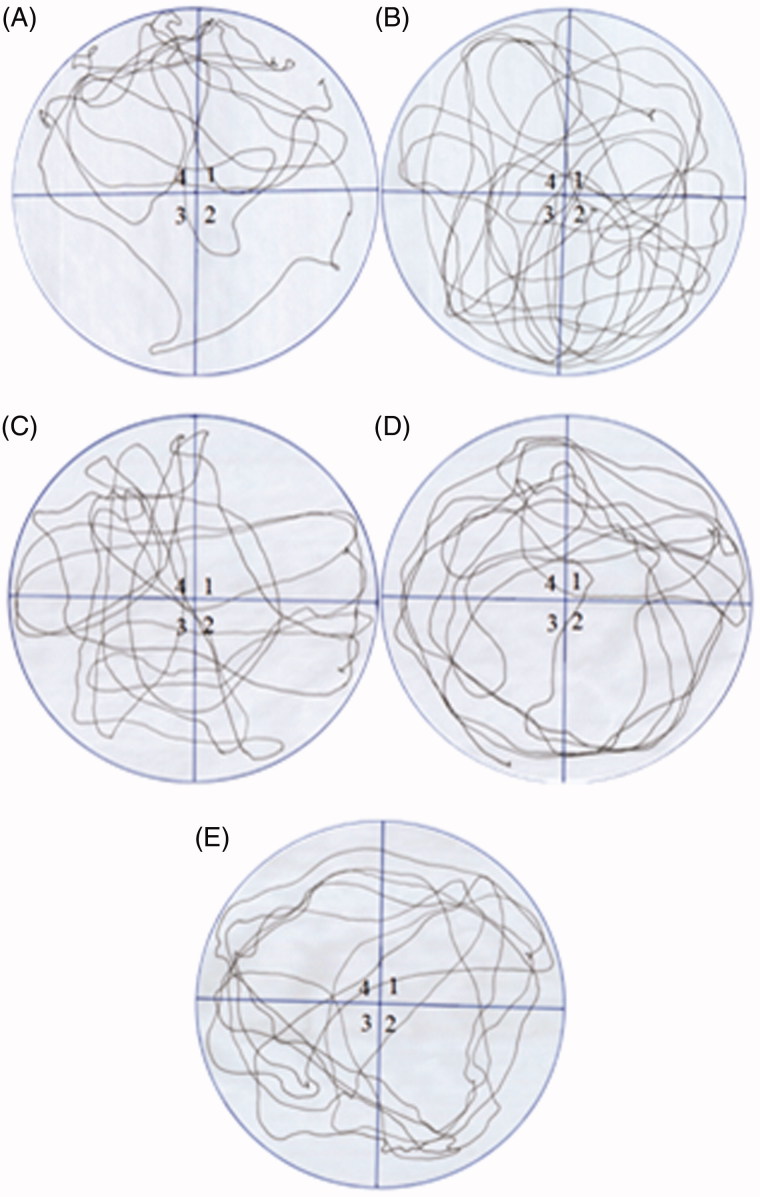
Comparison of the movement pattern of different treatment groups in probe trial. Quadrant 1 was the starting quadrant while quadrant 4 was the target quadrant. (A) Movement pattern of the vehicle control group. (B) Movement pattern of the fructose group animals (treated with d-fructose 1000 mg/kg (i.p.) plus 10% in drinking water). (C), (D) and (E) are movement pattern of rats treated with GLA 1, 5 and 15 mg/kg, respectively.

The fructose group showed significant decline in the time spent in target quadrant as compared to vehicle control (Figure 3, [*F*(4, 27) = 3.25, #*p* < 0.05]). The GLA treatment showed dose-dependent decline in the time spent in target quadrant. The effect was significant only at the lowest tested dose of 1 mg/kg as compared to fructose group [*F*(4, 27) = 4.01, *p < 0.05]. The movement patterns of representative rats from each group also showed similar results (Figure 4).

##### Number of crossings through platform position

**Figure 5.**
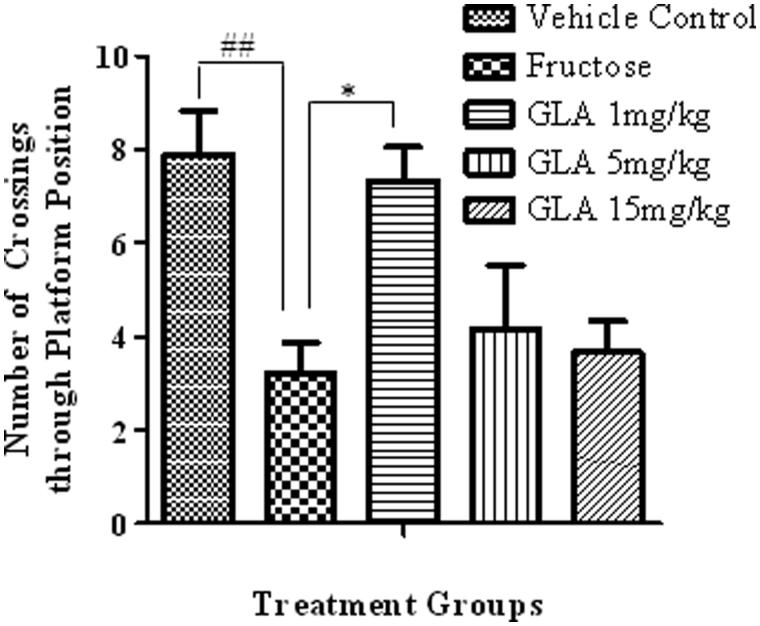
Effect of GLA on the number crossings through platform position in the probe trial. The data are expressed as mean ± SEM (*n* = 6). The fructose treatment has significantly reduced the number of crossing through platform position, which was significantly reversed by GLA (1 mg/kg).

In probe trial, the fructose-treated animals exhibited significant decline in the number of crossing through platform position as compared to vehicle-treated rats (Figure 5, [*F*(4, 27) = 5.91, ##*p* < 0.01]). The GLA treatment reversed the effect of fructose, which was significant only at 1 mg/kg [*F*(4, 27) = 4.32, *p < 0.05] and nonsignificant at 5 and 15 mg/kg.

##### Latency to enter the target quadrant

**Figure 6.**
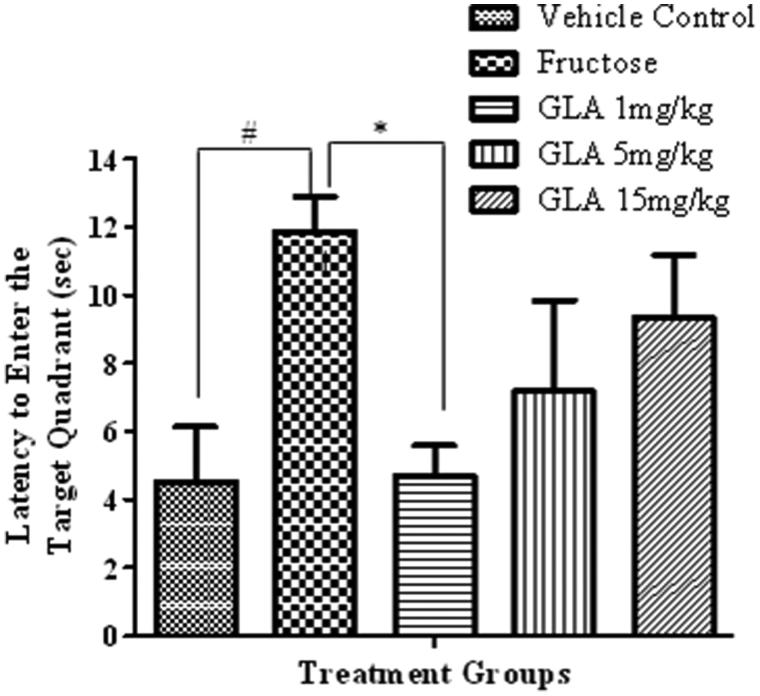
Effect of GLA on the latency to enter target quadrant in probe trial. The data are expressed as mean ± SEM (*n* = 6). The fructose treatment has significantly increased the latency to enter target quadrant, which was significantly reversed by GLA (1 mg/kg).

In probe trial, the fructose-treated animals exhibited significant increase in the time required to reach target quadrant as compared to saline-treated rats (Figure 6, [*F*(4, 27) = 2.99, #*p* < 0.05]). The GLA treatment reversed this effect of fructose, which was significant only at lowest tested dose of 1 mg/kg [*F*(4, 27) = 2.13, *p < 0.05].

### Locomotor activity test

**Figure 7.**
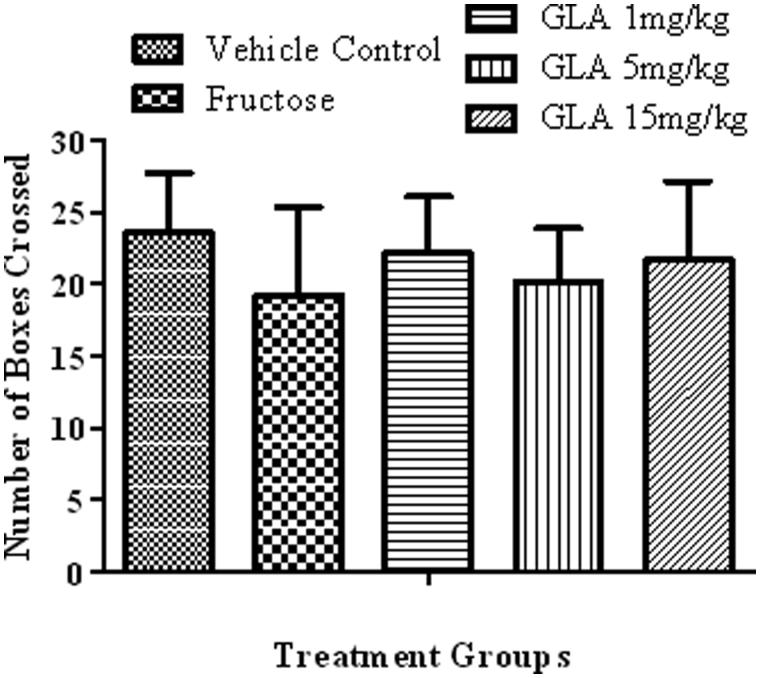
Effect of the various treatments on the locomotor activity of rats. The data are expressed as mean ± SEM (*n* = 6). None of the aforementioned treatment showed significant alterations.

Neither fructose [1000 mg/kg (i.p.) plus 10% in drinking water] nor GLA treatment (1, 5 or 15 mg/kg) showed significant alteration in the locomotor activity of rats (Figure 7).

### HbA1c measurement

**Figure 8.**
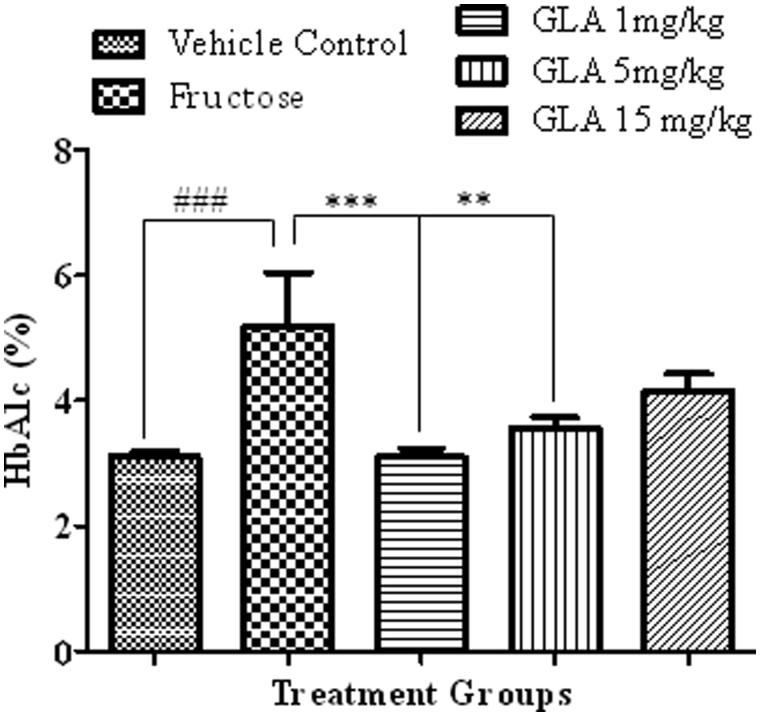
Effect of GLA on the HbA1c levels in the blood. The data are expressed as mean ± SEM of percentage HbA1c levels (*n* = 6). The fructose treatment has significantly increased the HbA1c levels, which was significantly reversed by the GLA (1 and 5 mg/kg).

The HbA1c levels of fructose group (animals d-fructose 1000 mg/kg (i.p.) plus 10% in drinking water) were found significantly higher as compared to that of the vehicle control group (Figure 8, [*F*(4, 26) = 8.76, ###*p* < 0.001]). The GLA administration significantly reversed the effect of d-fructose on the HbA1c level that was significant at 1 [*F*(4, 27) = 8.11, ***p < 0.001] and 5 mg/kg [*F*(4, 27) = 7.43, **p < 0.01] but nonsignificant at highest tested dose of 15 mg/kg.

### *In silico* study

**Figure 9.**
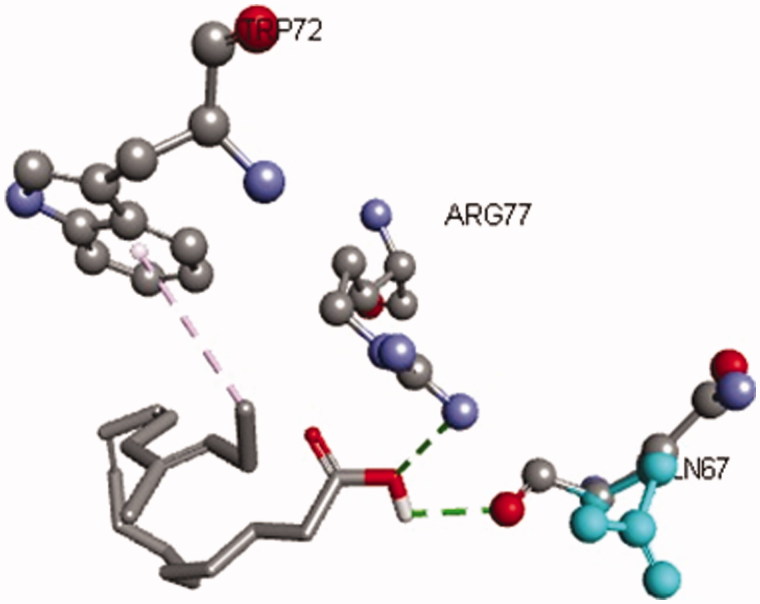
Interaction of GLA with active site residues of the RAGE receptor. The figure depicts hydrogen bonding (green dotted lines) of GLA with Arg77, and Gln67 residues and hydrophobic interaction (grey dotted lines) with Try72 of the binding site of RAGE.

The virtual screening showed that the CML underwent H-bonding with valine (Val78), leucine (Leu79) and glutamine (Gln67) residues of the binding site. Linolenic acid was seen to bind the active site residues of the receptor through H-bonding between the carboxylic acid oxygen atom and arginine (Arg77). The oxygen atom of glutamine (Gln67) of the receptor bind through H-bonding to the carboxylic group (-COOH) of GLA. A hydrophobic interaction was also seen between the Pi-electron of tryptophan (Trp72) and methyl group of GLA (Figure 9).

## Discussion

Ageing is associated with decline in various bodily faculties including cognition (Seidler et al. 2010). In this regard, among the various causes, the non-enzymatic glycation hypothesis of ageing received considerable importance. It states that the AGEs induced cross-linking of proteins which leads to age-associated decline in the cellular functions (Monnier 1988; Brownlee 1995). It has also been found that the level of AGEs increases with age, which co-relates with the development of various age-related pathologies like Alzheimer’s disease (Jono et al. 2002) and dementia (Yaffe et al. 2011). Hence, the process of glycation provides therapeutic target to alleviate AGE-mediated harmful effects observed in senescence.

Epidemiological data revealed that the consumption of polyunsaturated fatty acids is beneficial for memory and cognition (Kalmijn et al. 1997, 2004). GLA has been reported to cause various biological effects such as improvement of age-linked anomalies (Knauf et al. 2011). The notion was corroborated by our data which showed inhibition of AGE formation by GLA (Figure 1, IC_50_ 1.12 ± 0.05 μM). A review of the literature revealed that trapping reactive carbonyl intermediates is critical in limiting the formation of AGEs. This entrapment has been reported to be the prime target of AGE formation inhibitors and most likely underlie therapeutic potential of GLA as well. Notably, it was found to be more effective at low doses. The higher doses appear to enhance the formation of AGEs. In consistence with these results, high-fat diet was previously reported to enhance AGE formation (Li et al. 2005). In continuation of this outcome, the animal model of accelerated ageing (enhanced glycation) was developed (using high fructose diet) to assess the effect of GLA *in vivo*. Literature revealed that fructose accelerates glycation and production of AGEs (Farooqui 2013), which subsequently cause memory impairment (Yaffe et al. 2011; West et al. 2014). Our data also showed impairment in spatial learning of fructose-treated animals during acquisition phase (Figure 2(b)) as compared to control rats, which exhibited progressive decline in the escape latency (Figure 2(a)). In probe trial, the high fructose administration decreased the time spent in the platform quadrant (Figure 3), decreased the number crossing through platform position (Figure 5) and increased the latency to reach the target quadrant (Figure 6). Importantly, the GLA treatment (1, 5 or 15 mg/kg) significantly reversed all these parameters altered by d-fructose treatment. The aforementioned data are also supported by the movement patterns (Figure 4), which showed restricted movement of GLA-fed rats in the platform quadrant. It is important to note the lowest tested dose of GLA, i.e. 1 mg/kg was most effective in antagonizing the effect of fructose *in vivo*. This pattern is in conformity with the aforementioned AGE formation assay. The similarity of patterns among these assays (*in vitro* and *in vivo*) supports the notion that inhibition of AGE most likely underlies the memory enhancing effect of GLA. In the present study, the lack of effect at higher dose is supported by earlier reports linking memory decline with chronic high consumption of diet enriched with unsaturated or saturated fats (Greenwood & Winocur 1990; Winocur & Greenwood 2005). This outcome can possibly be explained in a way that GLA being lipid may itself be glycated at higher doses leading to increased AGE concentration. However, further work is required to delineate this perplexing outcome. Furthermore, the psychomotor stimulants can cause false-positive results in the MWM. Our data showed that GLA did not significantly alter the locomotor activity of the rodents (Figure 7). The probable reason for this outcome is the lack of interaction of GLA with the dopaminergic pathway (nigrostriatal) involved in locomotion. Hence, the antagonism of fructose effects by GLA in MWM was not an artefact.

In order to assess the glycation status of various treated groups, HbA1c level was measured in the blood of treated animals. Our data showed that the fructose treatment caused significant elevation of the HbA1c levels as compared to normal saline-treated rats (Figure 8). This is in line with the previous findings that manifested positive co-relation between blood HbA1c levels and high fructose diet (Hsieh et al. 2013). To the best of our knowledge, it is the first report attributing higher level of HbA1c in rats with the memory impairment. However, this association has been previously reported in human studies (Kerti et al. 2013). In consistency with aforementioned results (*in vitro* and *in vivo*), the GLA treatment has reversed this fructose-induced rise in HbA1c levels at lower doses (Figure 8). Hence, the dose of GLA appeared to be the critical factor in mediating its beneficial effect.

The effect of AGEs was reported to be mediated *via* RAGEs, which belongs to the super-family of immunoglobulin (Neeper et al. 1992). This receptor has an extracellular V shape domain which can bind numerous ligands including AGEs and β-amyloid (Koch et al. 2010; Yan et al. 2010). Interaction of AGEs with RAGE results in activation of various intracellular pathways. The most common signalling pathway activated by AGE–RAGE interaction is the induction of transcription factor NF-κB, which is considered to be the major contributor towards oxidative stress and nerve degeneration (Maczurek et al. 2008; Li et al. 2012). Importantly, the GLA has also been previously reported to alleviate oxidative stress (Nandakumar & Tan 2008) and inflammation *via* inhibition of NF-κB (Pacht et al. 2003; Calder 2006). This common target and aforesaid glycation modulation ability of GLA tempt us to virtually screen it against RAGE. Interestingly, our data showed the promising interaction of GLA with the active site of RAGE (Figure 9). Hence, it can be deduced that GLA not only has the ability to inhibit formation of AGE but can also intervene in the receptor-mediated deleterious effects of AGE. Furthermore, the pattern of aforementioned behavioural outcome is suggestive of competitive kind of interaction. Although these findings are preliminary, they provide new grounds to study the role of GLA–RAGE interaction in context of age-related effects on oxidative stress and learning and memory.

In conclusion, the GLA, at low doses, has the potential to ameliorate glycation-induced memory impairment. This effect appeared to be mediated *via* suppression of AGE production and interaction with RAGE. Based on these findings, GLA may be considered as a potential therapeutic candidate against glycation-mediated ailments such as memory impairment.
